# Brief report: Cause of death among people discharged from infective endocarditis related hospitalization—West Virginia, 2016–2019

**DOI:** 10.1002/clc.23812

**Published:** 2022-03-09

**Authors:** Zheng Dai, Gordon S. Smith, Brian Hendricks, Ruchi Bhandari

**Affiliations:** ^1^ School of Public Health West Virginia University Morgantown West Virginia USA

**Keywords:** cause of death, infective endocarditis, Medicaid, opioid use disorder

## Abstract

**Background and Objectives:**

Compare proportion of all‐cause and cause‐specific mortality among West Virginia Medicaid enrollees who were discharged from infective endocarditis (IE) hospitalization with and without opioid use disorder (OUD) diagnosis.

**Methods:**

The proportions of cause‐specific deaths among those who were discharged from IE‐related hospitalizations were compared by OUD diagnosis.

**Results:**

The top three underlying causes of death discharged from IE hospitalization were accidental drug poisoning, mental and behavioral disorders due to polysubstance use, and cardiovascular diseases. Of the total deaths occurring among patients discharged after IE‐related hospitalization, the proportion has increased seven times from 2016 to 2019 among the OUD deaths while it doubled among the non‐OUD deaths.

**Discussion and Conclusions:**

Of the total deaths occurring among patients discharged after IE‐related hospitalization, the increase is higher in those with OUD diagnosis. OUD is becoming a significantly negative impactor on the survival outcome among IE patients. It is of growing importance to deliver medication for OUD treatment and harm reduction efforts to IE patients in a timely manner, especially as the COVID‐19 pandemic persists.

## 1 INTRODUCTION

Opioid addiction has been a growing epidemic in the United States (US) reflected by the rapidly rising rates of opioid use and opioid overdose.^1^ The 2018 National Survey on Drug Use and Health (NSDUH) estimated that 2 million Americans were diagnosed with opioid use disorder (OUD)^2^ which is likely an underestimate due to self‐reported data and exclusion of populations at high risk of OUD in NSDUH. Only a fifth of them received specialty addiction treatment^2^; and among those accessing treatment, less than a third received medication for opioid use disorder (MOUD).^3^ Driven by surging synthetic opioids and polysubstance use with stimulants, alcohol, and benzodiazepines, there has been a drastic increase in the drug overdose deaths.^4^ However, we might only be seeing the tip of the iceberg of drug‐related deaths. While drug poisoning is the direct cause of death, opioid use and misuse is attributable to deaths caused by several co‐occurring medical conditions including lung disease, cancer, mental illness, HIV/AIDS, hepatitis, and infective endocarditis (IE).^3^ A recent US national study has found a high mortality rate among people hospitalized for IE, especially among patients with OUD.^5^ A North Caroline study found that drug use‐associated IE hospitalizations increased up to 12‐fold from 2007 to 2017.^6^ Therefore, estimating mortality after IE‐related hospitalization among the vulnerable Medicaid population is of great public health importance.

West Virginia (WV), a predominately rural state with low socioeconomic status, has been leading the nation's drug overdose mortality rate since 2014.^4^ According to the WV Department of Health Human Resources, WV Medicaid covered about 30% of the state population and included 71% of drug overdose decedents in the 12 months before their deaths. Thus, the WV Medicaid claims data could be a valuable resource to explore the impact of OUD on mortality. To date, little is known about the impact of OUD diagnosis on the survival among rural Medicaid patients discharged from IE‐induced hospitalization. The study aims to identify and compare proportion of all‐cause and cause‐specific mortality among WV Medicaid enrollees who were discharged from IE‐related hospitalization with and without OUD diagnosis.

## 2 METHODS

### 2.1 Data

This observational study used WV Medicaid administrative claims data to gather demographic and clinical information for the study population with at least 30 days of continuous enrollment from January 1, 2016, to December 31, 2019. Dual eligible individuals with Medicare were excluded since most of the claims went to Medicare. Age, gender, race/ethnicity, enrollment type, and enrollment periods were obtained from the eligibility file. Each record, extracted from facility and professional claim, contains primary diagnosis and up to a total of 25 diagnoses. International Classification of Diseases, Tenth Revision, Clinical Modification (ICD‐10‐CM) codes were used to identify IE‐related hospitalizations (I33, I38, and I39 from primary diagnosis) and OUD diagnosis (F11.1, F11.2, and F11.9 from a history of all diagnoses), among people aged 18–64 years. Hospitalization in WV Medicaid claims data was identified from a combination of place of service code (21) and room and board flag codes (1, 2, 3, or 4). Information of the deceased, including date of death (until December 31, 2019) and underlying cause of death coded in ICD‐10, was obtained from WV Vital Statistics, which was linked to the Medicaid data by an honest broker through deterministic approach with identifiable information removed before any analysis. The underlying cause of death is grouped by the 21 chapters of ICD‐10. The outcome of death was measured after discharge of patients from the first identified IE‐related hospitalization until the end of the study period (December 31, 2019). WV University Institutional Review Board granted this study exemption as this is a secondary data analysis used retrospective deidentified records (protocol number 2104295379), and an informed consent was not applied to this study.

### 2.2 Analysis

The annual death counts were grouped by the underlying cause of death defined by ICD‐10 chapters. The *χ*2 tests were used to compare demographics, number of IE‐related hospitalizations, and death counts between deceased with and without an OUD diagnosis. Fisher's exact tests were employed when the sample sizes of death counts were small. Between the OUD and non‐OUD decedents discharged from IE‐related hospitalization, the Student's *t*‐test was used to compare the number of IE‐related hospitalizations and Wilcoxon signed‐rank test was used to compare the age at death. Statistically significant level was set at 0.05.

## 3 RESULTS

Overall, 423 WV Medicaid beneficiaries had a total of 785 IE‐related inpatient admissions between 2016 and 2019. During this time, the number of IE hospitalizations increased by over a quarter (28%) each year. Among this population, 329 (77.8%) patients had ever had a diagnosis of OUD. The proportion of OUD among those who had IE inpatient stay increased from 71.0% in 2016 to 82.5% in 2019. The mean number of IE‐related hospital admissions was not significantly different between OUD and non‐OUD patients (1.88 vs. 1.78, *p* = .55).

By the end of 2019, almost a fifth (19.6%, *n* = 83) of all IE patients died. Of them, 59 (17.9%) had an OUD diagnosis compared to 24 (25.5%) without OUD (*p* = .11). Slightly over a third (36%) were under 35 years old, over a fifth (22%) were 36–45 years old and over a fourth (27%) were 46–55 years old. Males were about 10% more than females, and most decedents (92%) were non‐Hispanic White. The distributions of gender and race/ethnicity were not significantly different between OUD and non‐OUD IE decedents (*p* = .33 and .35, respectively). Among those discharged from IE‐related hospitalization, proportion of 1‐year mortality in OUD population increased from 1.9% in 2016 to 18.1% in 2018 and 14.2% in 2019, whereas it fluctuated among the non‐OUD population from 12.5% to 25%. Of the total deaths occurring among patients discharged after IE‐related hospitalization, the proportion of deaths increased seven times from 2016 to 2019 among the OUD while it doubled among the non‐OUD decedents. The top three underlying causes of death (shown in Table 1) among all IE patients were accidental drug poisoning (*n* = 24 [29%]), cardiovascular diseases (*n* = 19 [23%], and mental and behavioral disorders due to polysubstance use (*n* = 17 [20%]). Pathologies other than cardiology and drug‐related causes that led to death include neoplasms and diseases of the respiratory system, diabetes. Compared with non‐OUD decedents, deceased with OUD were more likely to die at younger age (median age 33 vs. 51.5 years, *p* < .01).

**Table 1:** Underlying cause of death among infective endocarditis cases

Disease group	ICD‐10‐CM	Number of deaths (%)
Accidental poisoning by and exposure to noxious substances	X41, X42, X44	24 (29%)
Diseases of the circulatory system	I25, I33.0, I35.8, I38, I42.9, I50, I51, I64	19 (23%)
Mental, behavioral, and neurodevelopmental disorders	F11.1, F11.9	17 (20%)
Pathologies likely related to infective endocarditis	A41.9, B37.6	<10
Other pathologies including neoplasms, diseases of the respiratory system, and so forth	B18.2, B20.1, C19, C22, C56, C73, E11.2, E88.0, G40.9, J18.9, J43.9, J44.9, J85.0, K74.6, K76.9, M86.9, N18.5, N18.9, W18, W69, X73	22 (27%)

Abbreviation: ICD‐10‐CM, International Classification of Diseases, Tenth Revision, Clinical Modification.

## 4 DISCUSSION

Findings from this study demonstrate the proportion of annual deaths in OUD population has increased seven times compared with deaths in non‐OUD population that has doubled during the same time among those discharged from IE‐related hospitalization, with the top three underlying causes of death being accidental drug poisoning, cardiovascular diseases, and mental and behavioral disorders due to polysubstance use. Substance use or misuse, including drug poisoning and associated mental disorders, and not any other cardiovascular pathologies, are the number one underlying cause of death among people discharged from IE‐related hospitalization. The number of hospital admissions was not significantly different between OUD and non‐OUD IE‐related patients. The majority of non‐OUD decedents who experienced IE hospitalization were older with cardiac and other medical complications.^7^ Although IE itself can be a fatal complication, the increasing number of deaths among this population is likely associated with increasing use of OUD (e.g., reusing or sharing contaminated syringes).^5^, ^8^ MOUD has been shown to be effective in reducing opioid use and preventing overdose.^1^ A study found that the receipt of medication for OUD is associated with 70% decrease in all‐cause mortality after injection drug use associated IE hospitalization; however, it is the long retention and close compliance with the treatment that save people's life, not just the initiation of medication for OUD.^9^ Strikingly, of the total deaths occurring among patients discharged from IE‐related hospitalization, those with OUD are dying at a much younger age compared to those IE decedents without OUD diagnosis (18.5 years younger at death), which is consistent with other studies.^8^ However, we might only see the tip of the iceberg of OUD as the underlying cause of death in WV due to its under‐diagnosis in rural patients.^10^ Given the high proportion of deaths among the OUD patients hospitalized for IE, inpatient OUD treatment is an important touchpoint that could save lives. Future studies need to explore the impact of COVID‐19 pandemic added to the long‐existing opioid pandemic on the survival outcome among people discharged from IE‐related hospitalization.

## 5 STRENGTHS AND LIMITATIONS

Strengths of the study include a successful linkage of Medicaid claims data and Vital Statistics to allow an in‐depth investigation into the specific causes of death among Medicaid enrollees discharged from IE hospitalization. In addition, the WV Medicaid claims data are rich in exploring the impact of OUD on mortality among an underserved population. This study has limitations, including small sample size and the possible lack of accuracy in death certificates for determining underlying cause of death. Nonetheless, WV uses a centralized medical examiner system that reports the highest percentage of drug intoxication deaths with drugs specified across the states.^11^ The study excluded persons who had dual eligibility with Medicare, which accounts for about 20% of overall Medicaid beneficiaries. The exclusion may bias the results to a relatively younger population. Also, disenrollment from Medicaid inevitably affects obtaining a complete picture of hospitalization history. The study used a minimum of 30 days continuous enrollment criteria to balance the sample size and capture of IE hospitalizations. In addition, the focus on WV Medicaid enrollees limits generalizability of study findings to this population.

## 6 CONCLUSIONS

Of the total deaths occurring among patients discharged after IE‐related hospitalization, the increase is higher in those with OUD diagnosis. This is despite the fact that OUD is underdiagnosed in patients with IE. Hence, it is a cause of grave public health concern, highlighting the importance of delivering MOUD treatment and harm reduction efforts in a timely manner, especially during the time COVID‐19 pandemic persists. Future research needs to include multiple causes of death analyses and expand OUD identification beyond claims data.

## CONFLICTS OF INTEREST

The authors declare no conflicts of interest.

## Data Availability

The data that support the findings of this study are available upon reasonable request made to the Office of Health Affairs. Restrictions apply to the availability of these data, which were used under approval of research request. Please contact Dr. Zheng Dai for further information.
